# China Tuberculosis Policy at Crucial Crossroads: Comparing the Practice of Different Hospital and Tuberculosis Control Collaboration Models Using Survey Data

**DOI:** 10.1371/journal.pone.0090596

**Published:** 2014-03-12

**Authors:** Xiaolin Wei, Guanyang Zou, John Walley, Jia Yin, Knut Lonnroth, Mukund Uplekar, Weibing Wang, Qiang Sun

**Affiliations:** 1 School of Public Health and Primary Care, The Chinese University of Hong Kong, Hong Kong, China; 2 China HSD Program, Nuffield Centre for International Health and Development, University of Leeds, Leeds, United Kingdom; 3 Nuffield Centre for International Health and Development, University of Leeds, Leeds, United Kingdom; 4 Global TB Programme, the World Health Organization, Geneva, Switzerland; 5 School of Public Health, Fudan University, Shanghai, China; 6 Center for Health Management and Policy, Shandong University, Jinan, China; Institut de Pharmacologie et de Biologie Structurale, France

## Abstract

**Background:**

Currently three hospital and tuberculosis (TB) collaboration models exist in China: the dispensary model where TB has to be diagnosed and treated in TB dispensaries, the specialist model where TB specialist hospital also treat TB patients, and the integrated model where TB diagnosis and treatment is integrated into a general hospital. The study compared effects of the three models through exploring patient experience in TB diagnosis and treatment.

**Methods:**

We selected two sites in each model of TB service in four provinces of China. In each site, 50 patients were selected from TB patient registries for a structured questionnaire survey, with a total of 293 patients recruited. All participants were newly registered uncomplicated TB cases without any major complications or resistance to first-line anti-TB drugs, and having successfully completed treatment. Diagnostic and treatment procedures were reviewed from medical charts of the surveyed patients to compare with national guidelines.

**Results:**

Specialist sites had the highest patient expenditure, hospitalization rates and mostly used second-line anti-TB drugs, while the integrated model reported the opposite. The median health expenditure was USD 1,499 for the specialist sites and USD 306 for the integrated sites, with 83% and 15% patients respectively having unnecessary hospitalization. 74% of the specialist sites and 19% of the integrated sites used second-line anti-TB drugs. Mixed results were identified in the two dispensary sites. One site had median health expenditure of USD 138 with 12% of patients hospitalized, while the other had USD 912 and 65% respectively.

**Conclusion:**

The study observed prohibitive financial expenditure and a high level of deviation from national guidelines in all sites, which may be related to the profit-seeking behavior of public hospitals. The study supports the integrated model as the better policy option for future TB health reform in China.

## Introduction

Tuberculosis is a global threat to public health, with around 12 million cases and 1 million deaths in 2011 [1]. In all countries, the national TB programs play the leading role in implementing of the World Health Organization (WHO) program. It is vital to include other health providers such as hospitals and private doctors in the treatment programme and ensure they follow the international standards of TB care [2]. Hospitals are usually the major providers of healthcare but they are often outside of the national TB programs. Hospitals may treat TB patients in a way different from the national TB guidelines. Substandard TB treatment, long delays and higher defaults had been reported in patients treated in big hospitals in Asian and African countries [3]–[5]. In China, public hospitals are the dominant health providers, and have decisive impacts on the TB program in terms of the key WHO TB control targets such as case detection and treatment success [6]. However, little is known in China regarding how hospitals collaborate with the TB control program.

China has the second highest TB burden in the world with 1.4 million patients [1]. The WHO Stop TB program (commonly called “DOTS”) was adopted in China since the early 90s. In 2000, the TB “DOTS” program including standardized diagnosis, treatments, monitoring and funding, demonstrated a success in the 13 project provinces with 36% decline of TB prevalence versus no change in the non-project provinces [7]. Then the TB program was expanded to all provinces in China in 2002. A semi-vertical system of TB control was established in China largely thanks to the TB program. This includes TB dispensaries at the national, provincial, prefecture and county/district levels. The county/district TB dispensary is the one-stop unit for all TB care, including clinical service such as TB diagnosis and treatment, and public health service such as data reporting, supply of first-line anti-TB drugs, training and coordination with hospitals. TB dispensaries at higher levels provide quality control for those at lower levels. There are three major TB control models in China.

### The dispensary model

This is the most prevalent model of TB practice in China. A TB dispensary is usually a department of the Centre for Disease Control (CDC). General hospitals take a passive role in this model, as they are responsible to refer TB suspects to dispensaries. Hospitals should not treat TB patients (except severe or complicated cases, referred by the TB dispensaries such as with drug resistance). TB dispensaries have the responsibility to trace cases referred to them by hospitals if they are not shown-up within three days. Free treatment is only provided to TB patients treated in TB dispensaries. The free treatment policy covers costs of the whole course of first-line anti-TB drugs, TB sputum smears and cultures, and X-ray examinations for the first and last months. The policy does not cover hospitalization costs, second-line anti-TB drugs and any other drugs or medical examinations during the TB treatment. TB cases are managed in the TB dispensaries during their treatment.

### The specialist model

This model is similar to the dispensary model except that a specialist TB hospital is located in the same district. The policy is that the specialist hospital should only treat severe TB cases, while uncomplicated cases should be treated in the TB dispensary under the DOTS program. The treatment cost in the specialist hospitals is not covered by the free treatment policy. In practice, a large number of uncomplicated cases are also treated in specialist hospitals. In this model, the specialist hospital has the responsibility of referring patients and TB suspects to TB dispensaries, but this role was often neglected [8]. This model is often found in big cities in northern China.

### The integrated model

This is a new development. A clinic provides TB care, under the free treatment policy, in a general hospital. The hospital is often the most popular local general hospital in the district, and is called as the TB ‘designated’ hospital. Patients in this model are diagnosed and treated under the TB program in the designated hospital. Other health providers including general hospitals should refer suspects and patients to the designated hospital. Under this model, the TB dispensary continues to provide the public health service aspects of TB control; such as training, mass education, case supervision, reporting and ensuring referred TB suspects arrived in the designated hospital. The model initiated in eastern China, and is practiced in Shanghai, Zhejiang, Jiangsu in 2002, and has expanded to a few sites in western provinces such as Guangxi.

China’s case detection rates was low in early 2000s, mainly due to the weak linkage between public hospitals and TB dispensaries [9]. China’s public hospitals were paid on fee-for-service basis, and relied heavily on user fees and drug margins for revenue [10].A national TB survey in 2000 showed that 91% of TB patients visited hospitals first, while only 25% of TB suspects in hospitals were referred to TB dispensaries [11].Patients treated in public hospitals were financially depleted and always severely delayedtheir diagnosis for TB [12]–[14].

The hospital TB collaboration has improved since 2005 thanks to the Internet based communicable disease reporting system developed after the Severe Acute Respiratory Syndrome (SARS) outbreak. All public hospitals are required to report TB suspects and cases online within 24 hours on the reporting system [15].In addition, the Ministry of Health published guidelines to improve the collaboration between general hospitals and TB dispensaries [16].All the above efforts substantially improved China’s case detection since 2005 [17]. However, profit-seeking behaviour in China’s public hospitals remains, which continued to cause high costs for TB patients [18], [19].

The current policy debates on the future of China’s TB control system. Rising of multi-drug resistance (MDR) TB and other complex TB cases pose a great clinical challenge to TB dispensaries [20].An argument posited that the specialist TB hospital has good clinical capacity, so it should treat all TB cases including drug sensitive patients. Another argument favoured the integrated model because people normally visit a general hospital for initial treatment. However, no supporting evidence is available for either argument. In response, this study aims to compare patient care experience within the different TB control models. We choose specifically drug sensitive patients because they account for the majority of TB patients. The study objective is to identify which model provides the best care for drug sensitive patients and with the least patient out-of-pocket expenditure.

## Methods

### Design of the study and settings

A facility level analysis was employed. Because the number of facilities for each model was not known, two sites of each model were purposively selected, with one urban and relatively rich site with gross domestic product (GDP) per capita over RMB20,000/USD2941, and another rural and relatively poor site with GDP per capita below RMB20,000. Due to the limited choices of sites with the integrated model, we selected one in Shanghai (east) and another in Guangxi (southwest). Both sites have started the integrated model for three years. The sites with specialist model were selected in Shandong province where the specialist model is more prevalent. The dispensary sites are widely available in China. We selected one in Zhejiang of eastern China and the other in Guangxi of western China. Each site consisted of a county/district where the basic TB management unit was located. ZD, SL and SC were relatively rich sites, while GP, SDC and GN were relatively poor sites. Full site names were blinded for confidentiality reasons. In the selected sites, general hospitals received less than 10% of their revenue from the government, while specialist hospitals received 20–30% from government. As public health agencies, TB dispensaries received around 85% of their revenues from the government.

### Patient recruitment

Only uncomplicated TB cases were recruited for comparison reasons because this group of patients accounts for the majority of total TB patients in China. Each site had around 100 uncomplicated TB cases registered in a year. We randomly selected 50 cases from its TB registry according to the inclusion criteria: 1) being new sputum smear positive or negative pulmonary TB patients, 2) been registered in 2007 and successfully completed treatment by August 2008, and 3) having no record of any serious co-morbidities such as diabetes, cardiovascular disease, hepatic disease or severe respiratory symptoms. All selected patients treated in the specialist hospitals were sensitive to first-line anti-TB drugs. Drug sensitivity tests were not available in other models. But all cases were unlikely to be drug resistant as they were recorded as ‘treatment successfully completed’. Participants were surveyed using a structured questionnaire adapted from the National TB Survey [11] which had been used in other similar settings [21], [22]. Questions covered patient social-economic status, care pathways, patient expenditure on TB care, and delays. Costs covered by health insurance schemes and the free treatment policy were often deducted automatically at the point of payment, so patients were not aware of the exact amount of costs covered by health insurance or the free treatment policy. Thus, we only collected patient out-of-pocket health expenditure. Patient charts provided information on medical examinations and drugs prescription.

### Ethics statement

All data were collected by a team of experienced researchers in 2008.Ethical approval was granted by the Ethical Committee for Health Policy Studies in Shandong University, China. All the subjects provided written informed consents. The dataset would be made available to researchers who formally apply through the corresponding author and comply with requirements of the Ethical Committee.

### Analysis

SPSS 14.0 (Chicago, USA) was employed for data analysis. Ordinal data analysis, one-way analysis of variance and chi-square test were used when appropriate. P = 0.05 was quoted consistently as the statistical significance level. Key study indicators included patient health expenditure, hospitalization, use of second-line anti-TB drugs, diagnostic and treatment delays. Median was used instead of the means to describe variables such as expenditure and delays whose distributions were highly skewed. According to the international standards of TB care, all uncomplicated patients should receive the treatment regimen of isoniazid, rifampicin, pyrazinamide, and ethambutol [23].We hypothesized that better TB and hospitals collaboration would result in better compliance of the national TB guideline, i.e., less use of second-line anti-TB drugs, less hospitalization, and less use of sophisticated examinations (not required in these cases), which leads to lower patient health expenditure and shorter delays. In another word, the better model would better comply with national TB guidelines and present better study indicators such as lower patient expenditure and shorter delays.

Diagnostic delay is the period from a patient’s first contact of healthcare until his/her TB diagnosis. According to the national guideline, TB should be diagnosed in dispensaries. In practice, TB is often diagnosed in hospitals. Here, the place of actual TB diagnosis is defined as where the patient was first informed of their TB diagnosis through results of sputum microscopy examination. Treatment delay is the period between TB diagnosis and the start of the TB chemotherapy. In this study, the TB treatment period was recorded in patient charts. The actual TB treatment period was usually longer than the recommended six months. Patients could not enjoy free treatment for the extra time exceeding the standard TB treatment time.

A household is defined as incurring catastrophic spending due to TB treatment if the patient reported health expenditure exceeding 40% of his/her household annual non-subsistence spending, which equals to total household spending minus food expenditure [24], [25]. This indicator was used to measure TB financial burden relative to income in different models.

## Results

Table 1 shows that all selected areas achieved over 85% cure rates in 2007. TB notification rates were 60–138 per 100,000 in the poor areas and 27–45 per 100,000 in rich areas. Within each model, rich areas had higher per capita government expenditure on TB control than poor areas.

**Table 1:** The general social economic situation and health financing for TB in the three models (2007).

	The Dispensary Model		The Specialist Model		The Integrated Model	
	ZD	GP	SL	SDC	SC	GN
Population	1010,000	160,000	1080,000	1200,000	750,000	6950,000
Per capita GDP (RMB)	26,120	17,545	47,586	13,369	88,785	15,774
TB expenditure per person (RMB)	0.52	1.54	0.42	0.18	0.99	0.42
Notification rate of TB patients (per million)	447	1,387	269	602	322	999
Reported cure rate of new smear positive TB cases (%)	93.4	90.6	89.1	99.3	88.5	89.8

1 USD = 6.8 RMB.

ZD, GP, SL, SDC, SC and GN are names of the selected sites in each collaboration model.

In total, 293 patients were surveyed. Table 2 shows the number of outpatient and inpatient charts collected in each site and the proportion of those available. In total, 250 outpatient charts and 88 inpatient charts were collected. Patients of the six sites shared similar demographic characters. Their mean age ranged from 42 to 53 years old, and 60% were male. Smear positive cases accounted for around half of patients and no significant differences were identified among models or sites (P = 0.16). The illiteracy rate was the highest in GP site of the dispensary model and SDC site of the specialist model (P<0.05). The majority of patients in the dispensary and specialist sites were farmers. Over 75% of patients were entitled to a health insurance program, i.e., basic employee and resident health insurance in urban areas or new cooperative medical insurance scheme in rural areas.

**Table 2:** Participants in each site and the general information of the patient survey.

	The Dispensary Model			The Specialist Model			The Integrated Model		
	ZD	GP	Sub-total	SL	SDC	Sub-total	SC	GN	Sub-total
Patient outpatient/inpatient charts	50/30	49/6		9/20	46/32		48/0	48/0	
Patients survey	51	49	100	44	46	90	50	53	103
Age	56	49	53	41	50	46	41	42	42
Male,N(%)	33(65)	29(59)	62(62)	28(64)	34(74)	62(69)	32(64)	33(62)	65(63)
Married,N(%)	42(82)	37(76)	79(79)	29(66)	29(63)	58(64)	31(62)	41(77)	72(70)
Illiteracy,N (%)†	9(18)	15(31)	24(24)	3(7)	14(30)	17(19)	1(2)	3(6)	4(4)
Farmer,N (%)‡	30(59)	37(76)	67(67)	15(34)	36(78)	51(57)	1(2)	23(43)	24(23)
With medical insurance,N (%)	45(88)	49(100)	94(94)	36(82)	46(100)	82(91)	35(70)	42(79)	77(75)
Per capita annual income (M, RMB) §	5,000	1,533	3,000	3,000	1,708	2,167	12,250	3,500	6,400
Borrowed money due to TB care, N (%)£	11(22)	22(45)	33(33)	20(46)	31(67)	51(57)	11(22)	18(34)	29(28)
Smear positive (%)	31(61)	29(59)	60(60)	19(43)	29(63)	48(53)	33(69)	20(42)	53(51)

1 USD = 6.8 RMB, M =  Median.

† GP was significantly higher than SL (χ^2^ = 8.409, P = 0.004), SC (χ^2^ = 14.952, P<0.001) and GN (χ^2^ = 10.908, P = 0.001), SDC was significantly higher than SL (χ^2^ = 8.187, P = 0.004), SC(χ^2^ = 14.693, P<0.001) and GN (χ^2^ = 10.627, P = 0.001).

‡ GP was significantly higher than SL (χ^2^ = 16.134, P<0.001) and GN (χ^2^ = 7.825, P = 0.005). SDC was significantly higher than SL (χ^2^ = 17.868, P<0.001) and GN (χ^2^ = 9.225, P = 0.002). ZD (χ^2^ = 37.013, P<0.001), SL (χ^2^ = 16.370, P<0.001) and GN (χ^2^ = 26.889, P<0.001)were significantly higher than SC.

§ Significantly difference was found among six sites (F = 11.907, P<0.001). SC was significantly higher than ZD (P = 0.015), GP (P<0.001), SL (P = 0.001), SDL (P<0.001) and GN (P<0.001). ZD was significantly higher than GP (P = 0.005) and SDC (P = 0.0026). SL (P = 0.042) and GN (P = 0.007) were significantly higher than GP.

£ SDC was significantly higher than ZD (χ^2^ = 20.685, P<0.001), SC (χ^2^ = 20.059, P<0.001) and GN (χ^2^ = 11.009, P<0.001).

### Patient care pathway and TB diagnosis

On average, a patient visited 3 health providers throughout the whole course of TB diagnosis and treatment, ranging from 4 in the specialist sites to 2.2 in the integrated sites (P<0.001). Hospitals were the major place for TB diagnosis. 37% of patients in the dispensary sites were diagnosed in general hospitals, while 56% in the specialist sites were diagnosed in specialist hospitals. Mixed results were found in the dispensary sites, with 59% patients in the ZD site diagnosed in the general hospitals versus only 14% in the GP site. Over 73% of patients received computerised tomography scan in the specialist sites and the ZD site of the dispensary model (Table 3).

**Table 3:** Health care seeking behavior of TB patients in the three models.

	The Dispensary Model			The Specialist Model			The Integrated Model		
	ZD	GP	Sub-total	SL	SDC	Sub-total	SC	GN	Sub-total
Patients survey	51	49	100	44	46	90	50	53	103
Usage rate of CT, N (%)†	37(72.5)	2(4.1)	38(38.0)	41(93.2)	29(63.0)	70(77.8)	26(52.0)	9(17.0)	35(34.0)
Usage rate of second-line anti-TB drugs,N(%)‡	34(66.7)	8(16.3)	42(42.0)	37(84.1)	30(65.2)	67(74.4)	9(18.0)	11(20.8)	20(19.4)
Health providers visited per patient (M) §	3	2	2	4	4	4	2	2	2
Diagnosis place									
Community health centre/Township hospital,N(%)	1(2.0)	0	1(1.0)	1(2.3)	0	1(1.1)	0	0	0
General hospital,N(%)	30(58.8)	7(14.3)	37(37.0)	2(4.5)	6(13.1)	8(8.9)	4(8.0)	3(5.7)	7(6.8)
County TB dispensary/Integratedhospital,N(%)	20(39.2)	42(85.7)	62(62.0)	6(13.6)	25(54.3)	31(34.4)	46(92.0)	50(94.3)	96(93.2)
TB special hospital,N(%)	NA	NA	NA	35(79.6)	15(32.6)	50(55.6)	NA	NA	NA

M =  Median.

† SL was significantly higher than ZD (χ^2^ = 6.844, P = 0.009), GP (χ^2^ = 74.037, P<0.001), SDC (χ^2^ = 11.819, P = 0.001), SC (χ^2^ = 19.388, P<0.001) and GN (χ^2^ = 55.892, P<0.001). ZD was significantly higher than GP (χ^2^ = 49.242, P<0.001) and GN (χ^2^ = 32.534, P<0.001). SDC was significantly higher than GP (χ^2^ = 37.521, P<0.001) and GN (χ^2^ = 22.093, P<0.001). SC was significantly higher than GP (χ^2^ = 28.015, P<0.001) and GN (χ^2^ = 14.064, P<0.001).

‡ ZD was significantly higher than GP (χ^2^ = 25.997, P<0.001), SC (χ^2^ = 24.458, P<0.001) and GN (χ^2^ = 22.319, P<0.001). SL was significantly higher than GP (χ^2^ = 42.627, P<0.001), SC (χ^2^ = 40.911, P<0.001) and GN (χ^2^ = 38.580, P<0.001). SDC was significantly higher than GP (χ^2^ = 23.631, P<0.001), SC (χ^2^ = 22.144, P<0.001) and GN (χ^2^ = 20.065, P<0.001).

§ Significant difference was found among six sites (F = 22.386, P<0.001). SL was significantly higher than ZD (p = 0.002) and GP (P<0.001). SDC was significantly higher than ZD (P<0.001) and GP (P<0.001). ZD was significantly higher than SC (P<0.001) and GN (P<0.001).

Although all patients were under the national TB program, a high proportion was treated with second-line anti-TB drugs. We found that 42% in the dispensary sites, 74% in the specialist sites and 19% in integrated sites had used second-line anti-TB drugs. In the specialist sites, 29 patients in the SL site and 9 patients in the SDC site received second-line drugs throughout their TB treatment. Commonly used second-line anti-TB drugs were quinolones (most commonly levofloxacin, but also ciprofloxacin and gatifloxacin), protionamide and p-aminoslicylic acid.

### Patient health expenditures and hospitalization

Patients of the integrated sites reported lower median health expenditure (RMB 2,080/USD 306) than those in the specialist sites (RMB 10,190/USD1,499, P<0.001). In the dispensary model, the ZD site had significant higher health expenditure (RMB 6200/USD 912) compared with the GP site (RMB 935/USD 138, P<0.001). In the specialist model sites and the ZD site of the dispensary model, high patient health expenditures were observed during the period between TB diagnosis and treatment. During the TB treatment period, patient expenditure was found the highest in the SDC site of the specialist model, followed by the ZD site in the dispensary model and the GN site in the integrated model, and then other sites (Table 4).

**Table 4:** Patient health expenditure for TB care in three models.

	The Dispensary Model			The Specialist Model			The Integrated Model		
	ZD	GP	Sub-total	SL	SDC	Sub-total	SC	GN	Sub-total
Patient survey	51	49	100	44	46	90	50	53	103
Total health expenditure(M, RMB)	6,200	935	2,380	10,415	9,403	10,190	1,550	2,600	2,080
Health expenditure before diagnosis (M, RMB) †	185	80	100	292	300	300	78	80	80
Health expenditure between TB diagnosis and DOTS treatment(M, RMB) ‡	2,000	0	0	8,300	1,515	5,450	0	0	0
DOTS treatment expenditure(M, RMB) §	2,000	600	1,160	500	2,700	2,200*	1,080	2,000	1,300
TB catastrophic expenditure ratio, N (%)£	31(60.8)	21(42.9)	52(52.0)	39(88.6)	36(78.3)	75(83.3)	15(30.0)	23(43.4)	38(36.9)
Proportion of household income as total health expenditure(%)	29.7	26.0	28.8	73.2	153.9	98.6	6.3	14.9	9.5

1 USD = 6.8 RMB, M =  Median.

* 29 patients of SL and 9 patients of SDC who were never treated in the TB dispensary, so they were excluded from the calculation of DOTS treatment expenditure. They were treated in the specialist hospital with a median treatment period of 209 days and the median health expenditure of RMB 11,985.

† Significant difference was found among six sites (F = 19.859, P<0.001). ZD was significantly higher than GP (P<0.001), SC (P<0.001) and GN (P = 0.001). SL was significantly higher than GP (P<0.001), SC (P<0.001) and GN (P<0.001). SDC was significantly higher than GP (P<0.001), SC (P<0.001) and GN (P<0.001).

‡ Significant difference was found among ix sites (F = 18.408, P<0.001). ZD was significantly higher than GP (P = 0.002), SC (P<0.001) and GN (P<0.001). SL was significantly higher than GP (P<0.001), SC (P<0.001) and GN (P<0.001). SDC was significantly higher than GP (P = 0.026), SC (P = 0.011) and GN (P = 0.015).

§ Significant difference was found among six sites (F = 3.7328, P = 0.011). ZD (P<0.001) and GN(P<0.001) were significantly higher than GP. SDC was significantly higher than GP (P = 0.001) and SC (P = 0.028).

£ ZD was significantly higher than SC (χ^2^ = 9.647, P = 0.002). SL was significantly higher than ZD (χ^2^ = 9.450, P = 0.002), GP (χ^2^ = 21.223, P<0.001), SC (χ^2^ = 32.918, P<0.001) and GN (χ^2^ = 21.335, P<0.001). SDC was significantly higher than GP (χ^2^ = 12.391, P<0.001), SC (χ^2^ = 22.408, P<0.001) and GN (χ^2^ = 12.432, P<0.001).

One third of patients borrowed money for TB care, while 56% of all patients incurred catastrophic spending due to TB treatment. Patient health expenditure for TB care accounted for 44% of their annual household income, ranging from 73% in the specialist sites to 15% in the integrated sites. Patients who borrowed money to treat TB were more likely to incur catastrophic expenditure (P<0.001). But no correlation was found between patients who borrowed money or who incurred catastrophic expenditure and who did not have health insurance (P = 0.881 and P = 0.820, respectively).

Patient inpatient expenditure accounted for the majority (68%) of their total health expenditure. In total, 44% were hospitalized. The specialist sites reported the highest hospitalization rates (83.3%, P<0.001), the highest median inpatient expenditure (RMB 8,900/USD 1,309, P<0.001) and inpatient days (35 days, P = 0.001), while the integrated sites reported the lowest (Table 5). Big differences were found within the two dispensary sites: the ZD site had higher hospitalization rates than the GP site (65% vs. 12%, P<0.001). In the specialist sites and the ZD site, over 80% of patients were hospitalized in the period between TB diagnosis and TB treatment.

**Table 5:** Hospitalization of TB patients in the three models.

	The Dispensary Model			The Specialist Model			The Integrated Model		
	ZD	GP	Sub-total	SL	SDC	Sub-total	SC	GN	Sub-total
Patient survey	51	49		44	46	90	50	53	
Total hospitalization,N (% of total patients) †	33(64.7)	6(12.2)		40(90.9)	35(76.1)	75(83.3)	8(16.0)	7(13.2)	
Inpatient days (M) ‡	19	12		35	30	35	15	20	
Inpatient expenditure(M, RMB)	5,030	3,815		9,028	8,554	8,900	4,010	6,000	
Hospitalization between TB diagnosis and DOTS treatment,N (% of total hospitalization) §	30(58.8)	3(6.1)		37(84.1)	23(50.0)	60(66.7)	3(6.0)	2(3.8)	
Inpatient days (M)	19	17		35	30	33	15	18	
Inpatient expenditure(M, RMB)	4,905	8,597		9,150	6,450	7,960	2,050	4,277	

1 USD = 6.8 RMB, M =  Median.

† ZD was significantly higher than GP (χ^2^ = 29.910, P<0.001), SC (χ^2^ = 24.836, P<0.001) and GN (χ^2^ = 29.122, P<0.001). SL was significantly higher than ZD (χ^2^ = 9.114, P = 0.003), GP (χ^2^ = 57.389, P<0.001), SC (χ^2^ = 52.556, P<0.001) and GN (χ^2^ = 58.115, P<0.001). SDC was significantly higher than GP (χ^2^ = 39.420, P<0.001), SC (χ^2^ = 34.980, P<0.001) and GN (χ^2^ = 39.862, P<0.001).

‡ Significant difference was found among six sites (F = 4.034, P = 0.004). SL was significantly longer than GN (P = 0.028).

§ ZD was significantly higher than GP (χ^2^ = 31.392, P<0.001), SC (χ^2^ = 32.025, P<0.001) and GN (χ^2^ = 36.975, P<0.001). SL was significantly higher than ZD (χ^2^ = 7.255, P = 0.007), GP (χ^2^ = 57.495, P<0.001), SDC (χ^2^ = 11.761, P = 0.001), SC (χ^2^ = 58.385, P<0.001) and GN (χ^2^ = 64.510, P<0.001). SDC was significantly higher than GP (χ^2^ = 22.979, P<0.001), SC (χ^2^ = 23.487, P<0.001) and GN (χ^2^ = 27.879, P<0.001).

### Delays

The sites in the integrated model reported the shortest diagnostic delay and total delay compared with the other two models (Table 6). Treatment delay was shorter in the integrated sites compared with the ZD site in the dispensary model (P<0.01).

**Table 6:** Delays of TB patients in three models.

	The Dispensary Model			The Specialist Model			The Integrated Model		
	ZD	GP	Sub-total	SL	SDC	Sub-total	SC	GN	Sub-total
Patient survey	51	49	100	44	46	90	50	53	103
Patient delay, 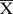 (M)	18(3)	41(7)	29(7)	11(5)	15(2)	13(3)	23(3)	24(7)	23(7)
Diagnostic delay, 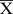 (M) †	26(2)	43(5)	35(4)	7(4)	39(12)	23(6)	7(1)	14(1)	11(1)
Treatment delay, 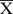 (M) ‡	13(9)	12(1)	12(1)	37(30)*	18(0)*	23(0)*	2(1)	2(1)	2(1)
Total delay, 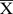 (M)	57(32)	96(33)	76(32)	57(44)*	70(33)*	66(36)*	32(14)	40(25)	36(17)

*29 patients of SL and 9 patients of SDC who were never treated in the TB dispensary, so they were excluded from the calculation of treatment delay and total delay. 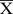 =  Mean, M =  Median.

† Significant difference was found among six sites (Z = 2.659, P = 0.025). SDC was significantly higher than SC (P = 0.040).

‡ Significant difference was found among six sites (Z = 3.651, P = 0.005). ZD was significantly higher than SC (P = 0.001) and GN (P = 0.001).

## Discussion

All patients recruited in this study were uncomplicated cases, i.e., new pulmonary TB cases without drug resistance or severe co-morbidities. According to international and domestic guidelines, they should be treated on an outpatient basis, and be given first-line anti-TB drugs. TB cases should be diagnosed by sputum smear and X-ray, not by sophisticated examinations such as computerized tomography scan [23], [26].Any hospitalization, use of second-line anti TB drugs and sophisticated examinations incurred by these uncomplicated TB cases were highly likely unnecessary. The study focused on uncomplicated cases because they accounted for the majority of TB patients in each model. Results and policy recommendation of this study do not affect specialist hospital’s role in treating complicated cases.

### Comparison among the three models

Different levels of deviations from national TB guidelines were observed. The specialist sites reported the worst study indicators. Nearly all patients used second-line anti-TB drugs. Patients in this model had the highest expenditure, and were largely hospitalized. The specialist hospitals should treat only complicated TB cases. However, they may tend to treat uncomplicated TB patients because specialist hospitals need to make a profit from treating patients. Another reason may be that patients choose to visit the specialist hospital for their high reputation in TB treatment. In practice, there were almost no TB patients referred from the specialist hospital to the TB dispensary [27]. One reason may be the weakness of government coordination because specialist hospitals are often at a higher governmental level than TB dispensaries. Another reason is that doctors in big hospitals often look down their peers in small clinics such as the TB dispensaries [6]. Surveys in Indian and Indonesia found similar problems in TB specialist hospitals, such as not following the Stop TB regimens [3], and hospitalizing uncomplicated cases [28].

Contrasting results were found in the two sites in the dispensary model. The ZD site had higher hospitalization rates and higher patient health expenditure, while the GP site reported the opposite. Other studies showed a mixed result in this model as well. Some reported improved case referrals between general hospitals and TB dispensaries after 2007 [29]–[31]. Others reported patients still experiencing long diagnostic delays and major expenditures in the same period [12], [21].

The integrated model showed better compliance of the TB national guideline, and presented the best results: the lowest patient health expenditure, hospitalization rate, and shortest delays. The two sites in the integrated model reported similar results despite their substantial differences in geographic locations. Several reasons were explored. First, patients with a persistent cough commonly visit general hospitals first [21], [22] in the integrated model sites, therefore they can be diagnosed and treated in the same place if found to have TB, which shortens patient care pathways. Second, we observed a swift transfer of TB patients from other departments to the TB clinic in the designated hospitals. A study investigating the integrated model in its early stage reported higher health expenditure in this model compared with the dispensary model. The study suggested that it was due to a lack of government input to the general hospitals [32]. In this study, we found the local governments provided financial support to set-up TB clinics in general hospitals. As well, the government supported the TB clinic’s running cost and regulated its operation. This financial support and supervision from the government is likely to be important to enhance the designated hospital’s willingness to run TB clinics.

### General challenges in all sites

Noncompliance to national guidelines was observed in all the three models. Overall, nearly half patients received hospitalization, and a significant proportion of patients were given second-line anti-TB drugs. In the dispensary and specialist model sites, over half patients were hospitalized after TB diagnosis. Early use of a second-line anti-TB drug may lead to the amplification of MRD TB [33].The national survey of drug-resistant TB in 2007 found that MDR TB was linked with inappropriate treatment in hospitals, especially specialist hospitals [34].Moreover, treatment in hospitals resulted in high financial burdens for TB patients. Despite the free TB treatment policy and the majority of patients being covered by medical insurance, the study found that patients had excessive financial burden. In most insurance schemes, coverage for outpatient care is generally a low percentage while the coverage for hospitalization was around 60–70% of the cost [35]. Importantly, the free TB treatment policy does not cover costs on auxiliary examinations, drugs and hospitalization. A large proportion of patients borrowed money for TB care, and incurred catastrophic expenditure due to TB care. TB expenditure in this study was higher compared with reports in other studies [36], because we selected patients treated in hospitals for comparison purposes.

China’s TB control is at crossroads after 20 years since the Stop TB program implementation. The enactment of the Law of Licensing Medical Practitioners called for gradually phasing out providing clinical care in CDC-based TB dispensaries as the CDC is an institute responsible for preventative care not clinical care. In addition, patients generally do not seek care in the TB dispensary as it has relatively low clinical capacity. Furthermore, TB prevalence rates in China has fallen substantially since the 1990s [7], [37]; thus, maintaining TB dispensary may not be cost-effective. The debate now focused on where clinical TB services should be best located.

Although the result of the study cannot be generalised across China, the implication, according to our hypothesis, is that the integrated model may demonstrate better hospital and TB program collaboration, and would be a better direction for reform. We also found that government financial support and regulation in the integrated model way are crucial. This model has an advantage because general hospitals are widely accessible in China. The most important challenge is how to protect patients from being exploited by China’s profit-oriented public hospitals [38], [39].Our study found that TB patients only accounted for a small proportion of total patients, so that the designated hospitals in the integrated models were less interested to profit from these patients. On the other side, the recent reforms on public hospitals have improved government investment for hospitals and limited the margins from drugs in hospital revenues [40]. This study is supportive of the integrated hospital model of TB collaboration as part of the on-going health reforms in China.

### Limitations

First, the integrated model and specialist model only operated in limited areas, so that we were not able to select sites with comparable economic development. Second, the study used a facility level analysis, thus it did not draw a representative sample of the three TB models in China. The two facilities in the specialist model and the integrated model showed similar indicator despite their differences in economic development. However, large differences were found between the two facilities in the dispensary model. This reflects great variations in the dispensary model similar to that found in the literature. Third, we only studied uncomplicated TB cases in each model. This study did not address the complicated cases treatment as these cases account for a small proportion of total TB cases and national treatment guidelines are less specific for these cases. Complicated TB cases are commonly treated in specialist hospitals, and this study did not intend to address this role of the specialist hospitals. Fourth, patient per capita income varies greatly across China. We here presented patient health expenditures in both absolute monetary terms, and relatively in relation to catastrophic spending. Patient information on income, expenditure and time may suffer from validity problems due to recall and reporting bias. The problem was minimized as the survey was taken within half a year after treatment completion. Information regarding TB treatment in hospitals and TB dispensaries, where possible, was collected from patient charts to cross-check potential recall problems. Fifth, we did not collect total costs because costs covered by health insurance were not available. Rural health insurance plans have a lower coverage rates than urban health insurance plans. However, 18–30% of participants in urban areas did not have health insurance coverage, which may offset the effects caused by different reimbursement rates between urban and rural areas. Lastly, we did not collect information regarding the proportion of budgets, financing and drug procurement of selected health institutions due to the sensitivity of this information.

## Conclusion

All three models of TB care carried high financial burdens for their patients. The integrated model reported the best outcomes. The specialist model sites reported the worst outcomes, having the highest rate of unnecessary hospitalization and consequent financial burden on patients. The sites in the dispensary model showed mixed results. The study is supportive of a policy shift towards integrating TB services into general hospitals.
